# The “Vestibular Eye Sign”—“VES”: a new radiological sign of vestibular neuronitis can help to determine the affected vestibule and support the diagnosis

**DOI:** 10.1007/s00415-023-11771-6

**Published:** 2023-05-23

**Authors:** Raed Farhat, Anan Abu Awad, Waleed Abu Shaheen, Diaa Alwily, Yaniv Avraham, Razi Najjar, Shlomo Merchavy, Saqr Massoud

**Affiliations:** 1 Department of Otolaryngology Head and Neck Surgery, Ziv Medical Center, Buqata, 1028 Safed, Golan Heights Israel; 2 Neurology Department, Ziv Medical Center, Safed, Israel; 3 Radiology Department, Ziv Medical Center, Safed, Israel

**Keywords:** Vestibular neuronitis (VN), Vestibular eye sign (VES), Benign paroxysmal positional vertigo (BPPV), Emergency department (ED)

## Abstract

**Introduction:**

Nystagmus is a valuable clinical finding. Although nystagmus is often described by the direction of its quick phases, it is the slow phase that reflects the underlying disorder. The aim of our study was to describe a new radiological diagnostic sign called “Vestibular Eye Sign”—VES. This sign is defined as an eye deviation that correlates with the slow phase of nystagmus (vestibule pathological side), which is seen in acute vestibular neuronitis and can be assessed on a CT head scan.

**Materials and methods:**

A total of 1250 patients were diagnosed with vertigo in the Emergency Department at Ziv Medical Center (ED) in Safed, Israel. The data of 315 patients who arrived at the ED between January 2010 and January 2022 were collected, with criteria eligible for the study. Patients were divided into 4 groups: Group A, “pure VN”, Group B, “non-VN aetiology”, Group C, BPPV patients, and Group D, patients who had a diagnosis of vertigo with unknown aetiology. All groups underwent head CT examination while in the ED.

**Results:**

In Group 1, pure vestibular neuritis was diagnosed in 70 (22.2%) patients. Regarding accuracy, VES (Vestibular Eye Sign) was found in 65 patients in group 1 and 8 patients in group 2 and had a sensitivity of 89%, specificity of 75% and a negative predictive value of 99.4% in group 1—pure vestibular neuronitis.

**Conclusion:**

VN is still a clinical diagnosis, but if the patient undergoes head CT, we suggest using the “Vestibular Eye Sign” as a complementary sign. As per our findings, this is a valuable sign on CT imaging for diagnosing the pathological side of isolated pure VN. It is sensitive to support a diagnosis with a high negative predictive value.

## Introduction

Nystagmus is a valuable clinical finding and it may be defined as a rhythmic, involuntary, rapid, oscillatory movement of the eyes [1]. Peripheral nystagmus can have two phases: slow and fast, or a combination of the two. The fast phase of nystagmus is normally directed away from the side of a destructive lesion, the slow phase sends the eye away from the preferred direction of gaze, and the corrective quick phase (a saccade) returns the eye to the visual target in a peripheral vestibular lesion. Although the fast stages of nystagmus are generally identified by their orientation, the slow phase is what reveals the underlying problem [2].

Peripheral nystagmus is transient, it is always due to unilateral, or if bilateral then asymmetrical, vestibular disease or stimulation. It is always suppressed by visual fixation. Failure of fixation suppression indicates cerebellar disease [3].

Nystagmus with central vertigo, on the other hand, is more likely to appear as “alternating” direction-changing nystagmus. When the patient looks to the right, the nystagmus will be rightward, and when the patient looks to the left, the nystagmus will be leftward [4].

It can be difficult for clinicians to appropriately categorize the nystagmus phase at times. Seeing the patient's eyes move swiftly in real time and determining which side is pathogenic and which is etiological might be difficult. In patients with acute vertigo, detecting nystagmus is a crucial diagnostic signal. Patients with peripheral abnormalities have a continuous direction of nystagmus, whereas those with central disorders have nystagmus that changes direction with or without gaze fixation [5].

Misdiagnosis of common peripheral vestibular disorders such as benign paroxysmal positional vertigo (BPPV) and vestibular neuritis results in inefficient therapy and resource overuse [6].

Over 90% of all vertigo causes are caused by peripheral vertigo. Ischemia of the central vestibular structures in the cerebellum, brainstem, or vestibular nuclei is the most prevalent cause of central vertigo, especially in elderly individuals with vascular risk factors. In younger individuals, acute demyelination, such as that seen in multiple sclerosis, is another rather prevalent cause of central vertigo [7]. Cerebellar haemorrhage, thiamine deficiency, and numerous autoimmune, viral, or metabolic conditions [8, 9] are all rare causes of isolated acute vestibular syndrome (AVS).

Vestibular neuritis (VN) is a condition that causes vertigo, nausea, and gait instability. It is hypothesized to be caused by inflammation of the vestibular component of the eighth cranial nerve. It is a harmless, self-limiting disease that usually lasts a few days, but it can take weeks or months for all vestibular symptoms to disappear [10]. This is a clinical diagnosis, and it is up to the physician to tell the difference between this benign self-limiting condition and other central nervous system causes, such as cerebrovascular syndromes [11].

However, because of the significant underlying aetiology of brainstem ischemia or infarction [5], the clinical diagnosis of central vertigo is critical. VN is a diagnosis of exclusion based on clinical, laboratory, and radiographic evaluations [12] because there are no confirmatory diagnostic tests. Even if a patient shows the typical pattern of spontaneous nystagmus seen in vestibular neuritis, brain imaging should be considered if the patient has an unusual headache, a negative head impulse test, severe unsteadiness, or no improvement after 1–2 days [13].

Guarnizo et al. [14] concluded that both non-contrast computed tomography and computerized tomography angiograms of the head and neck have low diagnostic yield for the detection of central causes of dizziness. However, they suggested that more research be done to determine the role of computerized tomography in the work-up of patients with isolated dizziness in the emergency department.

Vestibular Eye Sign (VES) is seen in head CT as an eye deviation in a horizontal gaze toward the affected vestibule, and it has never been mentioned in predicting the affected side of the vestibulopathy.

The aim of our study was to describe a new radiological diagnostic sign in vestibular neuronitis known as the "Vestibular Eye Sign" (VES).

## Patients and methods

### Patients

This study was a retrospective review of study patients who were diagnosed with vertigo and already have undergone CT in the ED in Ziv Medical Center between January 2010 and January 2022.

Both the study protocol and the use of data were approved by the Helsinki Committee of the Medical Center.

### Inclusion and exclusion criteria

#### Exclusion criteria

Patients with reports of unilateral hearing loss, history of external or middle ear problems, history of inner ear surgery, ototoxic drug intake, previous neurologic disorders, incomplete clinical data, and with a known aetiology, including trauma, prior eye globe deviation, migraine, and Meniere disease, were excluded from the study.

#### Inclusion criteria

**Fig. 1:**
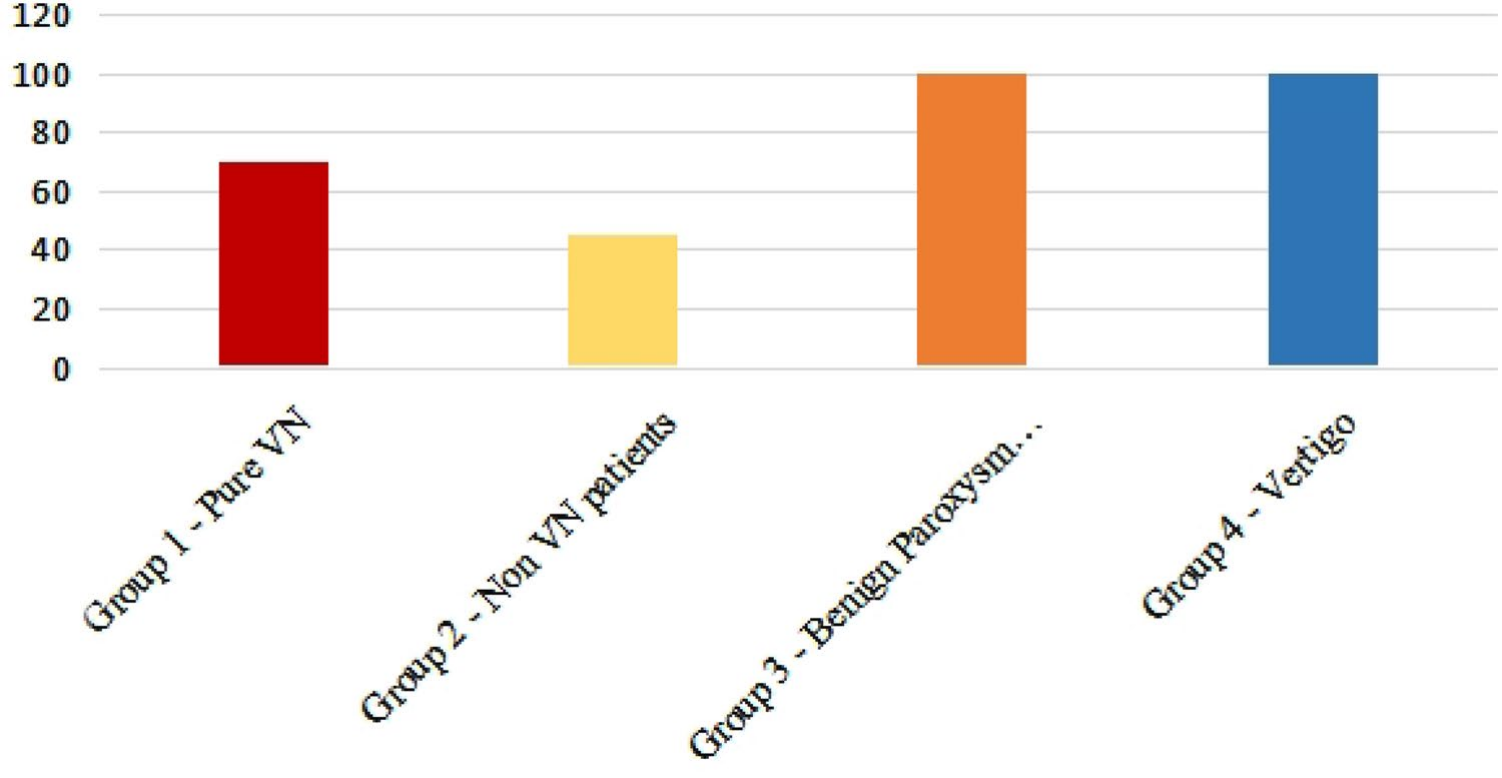
Number of patients in each group of the study

Patients were divided into 4 groups (1 + 2 with spontaneous nystagmus 3 + 4 with no spontaneous nystagmus) (Fig. 1).

*Group 1* “pure VN “: The diagnosis of Group 1—“Pure vestibular neuronitis” was based on the diagnostic criteria of Acute Unilateral Vestibulopathy according to the Bárány Society Classification of Vestibular Disorders [14] (Table 1).

**Table 1:** The diagnostic criteria of acute unilateral vestibulopathy according to the Bárány Society Classification of Vestibular Disorders [13]

Diagnostic criteria for “Acute Unilateral Vestibulopathy” Each of the following criteria have to be fulfilled
(A) Acute or subacute onset of sustained spinning or non-spinning vertigo (i.e., an acute vestibular syndrome) of moderate to severe intensity with symptoms lasting for at least 24 h
(B) Spontaneous peripheral vestibular nystagmus i.e., a nystagmus with a trajectory appropriate to the semicircular canal afferents involved, generally horizontal-torsional, direction-fixed, and enhanced by removal of visual fixation
(C) Unambiguous evidence of reduced VOR function on the side opposite the direction of the fast phase of the spontaneous nystagmus
(D) No evidence for acute central neurological symptoms or acute audiological symptoms such as hearing loss or tinnitus or other otologic symptoms such as otalgia
(E) No acute central neurological signs, namely no central ocular motor or central vestibular signs, in particular, no skew deviation, gaze-evoked nystagmus, or acute audiological signs16
(F) Not better accounted for by another disease or disorder

*Group 2* “non-VN aetiology”: those patients who were suspected to have VN but the diagnosis was ruled out in follow-up and had neurologic symptoms such as weakness, vision or hearing changes, altered level of consciousness, truncal ataxia, transient or vertical nystagmus, cranial nerve deficits or other changes in sensory and motor function favouring the presence of a central cause of vertigo such as cerebrovascular disease, neoplasm, multiple sclerosis or B12 deficiency Patients who had an MRI scan with neurological follow-up were included as an isolated group in the study named “Non-VN cause”.

*Group 3* patients who were diagnosed with benign paroxysmal positional vertigo (BPPV) who had head CT while in the ED with no central cause, with no spontaneous nystagmus.

*Group 4* patients who had a “vertigo” diagnosis in the ED and underwent head CT while in the ED with both normal otolaryngologic and neurological evaluation, with no spontaneous nystagmus.

All of the participants were subjected to history taking and otoscopy and complete neuro-otologic examination, including bedside vestibular examination, to differentiate peripheral versus central vestibular lesions. Groups 1 and 2 underwent cranial MRI with gadolinium enhancement.

The control group was formed from Groups 3 and 4 (with no spontaneous nystagmus).

### Methods

#### CT diagnosis criteria of the vestibular eye SIGN

CT scans of 315 serial patients presenting with symptoms of acute vertigo were reviewed.

Axial head scans were acquired with CT scanners with the slices parallel to the inferior orbitomeatal line or orbitomeatal line and slice thickness of no more than 6 mm through the orbit.

No instructions were given to the patients regarding eye closure or gaze during the scan, but the 3D reconstruction revealed that the pure vestibular neuritis group (Group 1) had closed eyes.

Three raters blinded to all clinical information independently assessed the orbits and the data were collected and interpreted by a neuroradiologist.

**Fig. 2:**
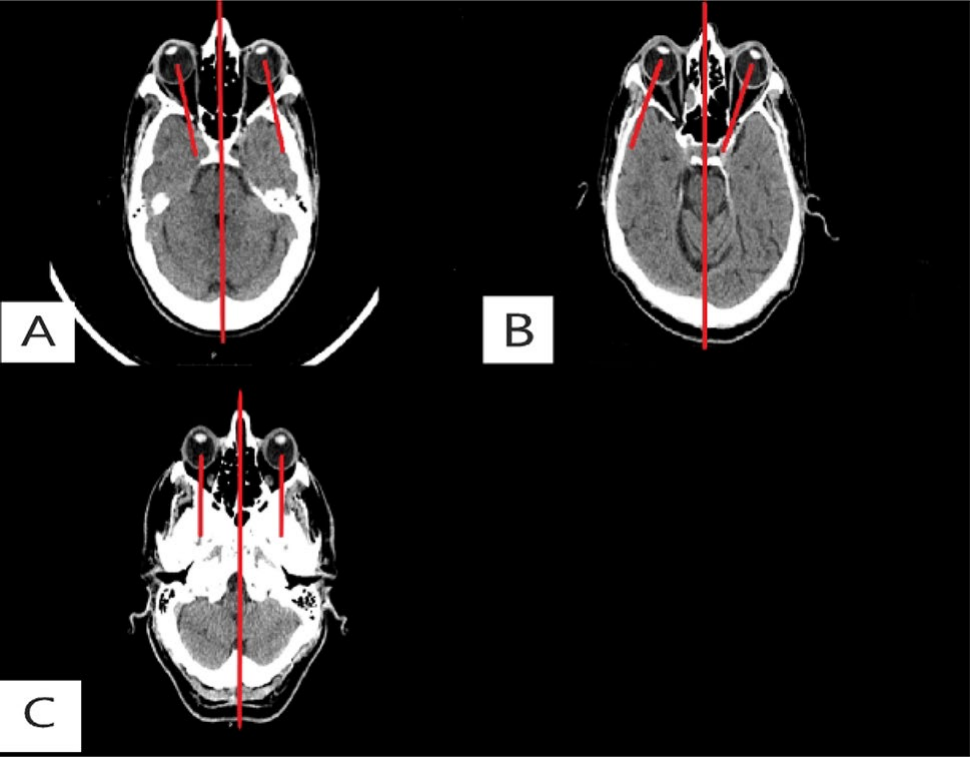
Non contrast head CT showing: **A** right vestibular neuronitis–nystagmus slow phase to the right VES Sign-rightward deviation. **B** Left vestibular neuronitis–nystagmus slow phase to the left VES Sign-leftward deviation. **C** A patient from group B (Non-VN etiology) with undeviated “intermedita” globe

Scans were classified as showing rightward globe, leftward globe, or undeviated indeterminate globe. Indeterminate scans were those where the axes were not visible. If there was movement of the ocular axes across the midline between slices, the scans were classified as undeviated (Fig. 2).

**Fig. 3:**
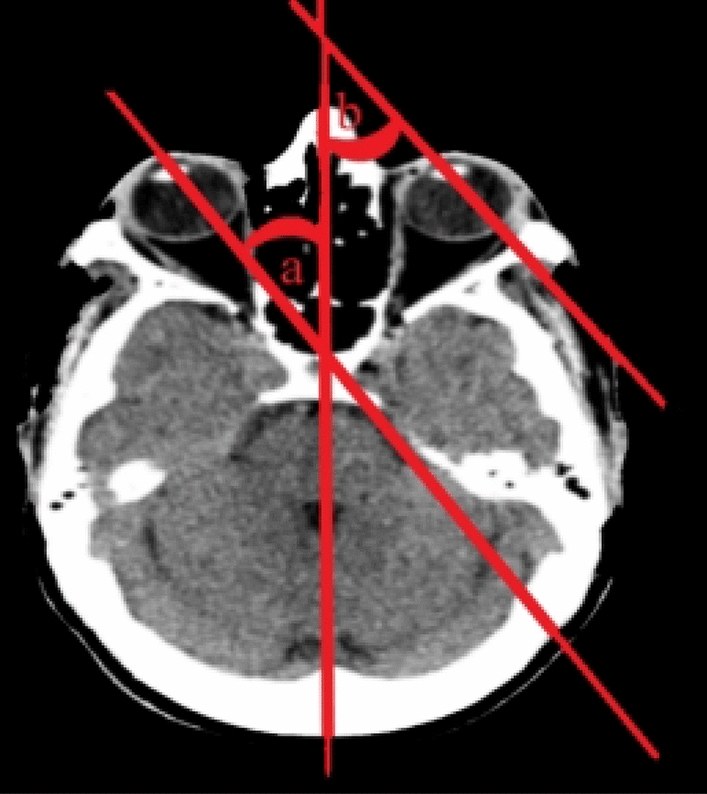
The angles formed by the intersection of the ocular axes of the right (**a**) and left (**b**) eye and the ‘line of best fit’ through the midline structures of the head were determined

The authors determined the horizontal deviation of eye-in-head position by the using the angle created by the ocular axis's junction with the “line of sight” greatest fit' through the head's midline structures (Fig. 3).

The researcher who carried out the measurements was blinded to the clinical signs of the patient. Horizontal deviations in the direction of positive values were classified on the ipsilesional side, whereas deviations to the contralesional values on the negative side. The values of the right and left eye deviations averaged for every person.

The raters were not given any instructions as to what degree of deviation constituted each of the gaze categories.

Independent measurement quantified the degree of eye deviation and gave an estimate of the threshold at which interpreters typically differentiated between deviated and undeviated eyes.

Consensus ratings (agreement of two out of three raters) and kappa statistics for interobserver agreement were calculated.

Vestibular lesion location was determined by clinical information with fast- and slow-phase nystagmus, which were noted in the patient examination and diagnosis.

The probability that eye deviation correctly predicted the pathological vestibular side was calculated.

### Statistical analysis

Data were analysed using SPSS version 25 (SPSS Inc. Chicago, IL, USA).

All continuous variables are presented as the mean and standard deviation, while all categorical variables are presented as frequencies and percentages.

The 2 × 2 contingency table was formulated to determine the sensitivity, specificity, positive predictive value, and negative predictive value of the “Vestibular Eye Sign” in head CT taking clinical findings and normal MRI as the gold standard.

## Results

We identified 1250 patients diagnosed with vertigo during the study period, of whom 315 fit the inclusion criteria. Of the 315 patients, 70 were in group 1, 45 were in group 2, 100 were in group 3 and 100 were in group 4. The mean patient age was 47.2 ± 17.5 years in group 1 and 65.2 ± 12.6 years in group 2, and the mean age was 64.75 ± 9.5 years in group 3 and 55.4 ± 10.3 years in group 4.

There were 187 (59.3%) female and 128 (40.7%) male patients.

**Fig. 4:**
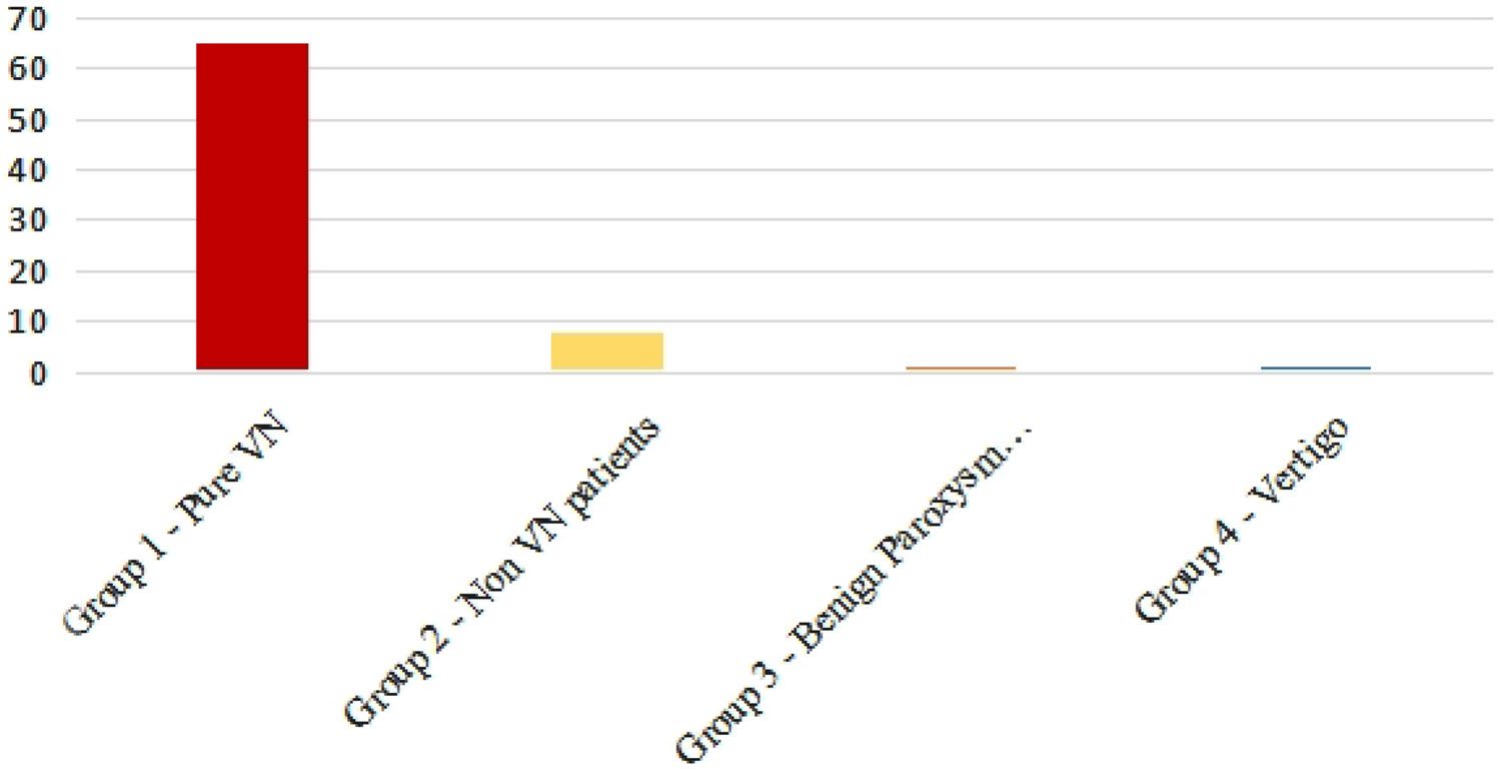
Number of positive VES in each group

VES was found in 65 patients in the pure VN group and 8 patients in the non-VN group and was not found in groups 3 and 4 (Fig. 4).

VES was found to have a sensitivity of 89%, specificity of 75%, positive predictive value of 15% and negative predictive value of 99.4% in group A—pure vestibular neuronitis (Table 2).

**Table 2:** Contingency table representing the “Vestibular Eye Sign” in Pure VS, Non VS and BPPV with Vertigo age-matched controls

VES evaluation	Pure VN (*n* = 70)	Non-VN (*n* = 45)	BPPV (*n* = 100)	Vertigo (*n* = 100)
Positive (*n*)	65	8	0	0
Negative (n)	5	37	100	100
Proportion of the positive VES Sign	92%	21%	0%	0%
Prediction of the affected Vestibule	97%			
Test parameters				
	***P* < 0.001**			
Sensitivity	**89%**	27%		
Specificity	75%	88%		
PPV	15%	8%		
NPV	**99.4%**	96.6%		

Bold values indicate statistically significant

Test parameters included: Sensitivity, Specificity, Positive Predictive Value (PPV) and Negative Predictive Value (NPV) calculated from pure VN and non-VN patients’ evaluations

(*n*) number of evaluations

The diagnostic data of VES in diagnosing pure vestibular neuritis compared to non-VN groups are shown in Table 3.

**Table 3:** Out of the 70 cases of pure vestibular neuritis, 65 patients had VES Sign while 5 cases case did not present with VES Sign

Groups	Positive Vestibular Eye Sign	No VES	Total	*P* value
Group 1: pure-VN	65 (17.11) [134.03]	5 (52.89) [43.36]	70	*P* < 0.001*
Group 2: non-VN aetiology	8 (11.00) [0.82]	37 (34.00) [0.26]	45	
Group 3: BPPV	0	0	100	
Group 4: Vertigo	0	0	100	

Bold value indicates statistically significant

^*^The result is significant at *P* < 0.05

The side of the eye deviation of the VES was not significantly different in groups A and B (Table 4).

**Table 4:** The side of VES distribution was not statistically different in Groups A and B

Study groups	Right side	Left side	*P* value
Group 1: pure VN	39 (36.63) [0.15]	31 (33.37) [0.17]	*P* value is 0.341*
Group 2: non-VN aetiology	3 (5.76) [1.32]	8 (5.24) [1.45]	
Group 3: BPPV	0	0	
Group 4: Vertigo	0	0	

Bold value indicates statistically significant

^*^The result is significant at *P* < 0.05

## Discussion

Based on the results of this study and the literature, we recommend using the "Vestibular Eye Sign" in conjunction with clinical data to diagnose acute vestibular neuronitis. This is a useful indicator on CT imaging for diagnosing the pathogenic side of isolated pure VN, according to our findings. It has a sensitivity of 89% and a specificity of 75%, as well as the potential to have a high negative predictive value of 99.4%.

Our study results confirm the importance of VN as a clinical diagnosis.

This is not the first time "Vestibular Eye Sign" has been described in the medical literature as a radiological sign for acute VN [15], but it has never been mentioned in predicting the affected side of the vestibulopathy.

If the clinical diagnosis is very probable for VN, the VES is sensitive in predicting the affected side of the vestibulopathy and in confirming this diagnosis, but if it was not found, the clinician should be doubtful and more open to other diagnoses despite the fact that the VES does not rule out VN.

We emphasize the fact that inappropriate CT usage can disclose incidental abnormalities that compromise the validity of the diagnosis and prompt useless, pointless, and even dangerous follow-up tests [16].

After a CT was performed, we evaluated our patients retrospectively and there was ocular lateral deviation.

The patient should next be examined to identify the side that is likely impacted by vestibular neuritis; however, according to our results and based on other articles, some individuals may have actually experienced a lateral medullary stroke [17, 18] or even a hemisphere stroke [19].

The clinical syndrome of sudden spontaneous vertigo (for several days) without any other neurologic or audiologic symptoms or signs is usually attributed to vestibular neuritis (VN) [20, 21], which is caused by viral or post-viral inflammation of the vestibular nerve.

Cerebellar stroke [22], also known as pseudo-VN, causes comparable clinical symptoms and findings. A cerebellar infarction linked with pseudo-VN can be readily missed on a CT scan [23].

Lee et al. [24] concluded that cerebellar infarction mimicking vestibular neuritis is more common than previously assumed and that early diagnosis and recognition of this indication is critical.

According to a large prospective study, approximately 11% of patients with isolated cerebellar infarctions experience isolated vertigo, and the majority of them (96%) had an infarct in the region of the medial branch of the PICA, which includes the nodulus [25].

Ischemia of the lateral medulla, which includes the vestibular nucleus, may be a prevalent source of isolated vascular vertigo since the vestibular nucleus is more sensitive to ischemia than other structures in the brainstem and cerebellum. Isolated vertigo can also be caused by lesions involving the flocculus or dorsal insular cortex [26].

Eye deviation on admission was related to substantial "anterior" circulation and more severe neurological impairments, according to S. Payabvash et al. [27].

In patients with acute unilateral cerebellar lesions, gaze-evoked nystagmus (GEN) may not only be a diagnostic indicator in patients with brainstem lesions but also signal to ipsilesionally localized destruction of midline and lower cerebellar structures [28], according to a study.

Vestibular strokes are frequently misdiagnosed due to clinical symptoms that resemble benign ear diseases. Because over 95% of ED dizziness patients do not have a stroke, diagnosing these cerebrovascular instances is extremely difficult.

Neuroimaging appears to be a natural answer; however, CT seems ineffective, and MRI is imperfect and too expensive to use on all patients who go to the ED with vertigo. This puts a premium on precise bedside diagnosis [29].

The following literature study can explain the meaning of the "Vestibular Eye Sign":

A- Eye deviation seen on CT scans seems to occur more frequently than what is seen during a clinical evaluation. This is possibly because fixation is removed when most patients close their eyes during CT scanning.
B- In the absence of any nystagmus in the light, the appearance of a unidirectional nystagmus with eye closure or in darkness, or an increase of nystagmus present in the light by eye closure or in darkness [30].
C- Because the patient has less time to move during the acquisition, fast scanners reduce motion artifacts. Faster rotation or more X-ray sources could help achieve this [31].

Our study limitations were that it was a retrospective, the use of a single-centre study, and the lack of a quantitative definition for eye deviation observed on admission CT. In addition, given that only the consensus interpretations of CT images were available in the trials public dataset, the interrater agreement cannot be determined; however, prior studies have reported an excellent interrater agreement for the determination of radiological eye deviation with a Kappa coefficient of 0.8.

Our study strengths were that only two prior researches on this specific topic [15, 32] and it is a new research typology.

In this case, discovering the limitations can be considered an important opportunity to present the need for further development in this area of study.

The use of a radiological "Vestibular Eye Sign" to distinguish posterior circulation stroke from vestibular neuritis has never been reported in the literature, but the goal of this research is to first describe this sign in the peripheral VN and then compare it to posterior circulation stroke in a separate study.

## Conclusion

Vestibular neuronitis is still a clinical diagnosis, but if the patient undergoes a head CT, we suggest using the “Vestibular Eye Sign” as a complimentary sign and may aid in the localization of the affected vestibular side. As per our findings, this sign is a valuable sign on CT imaging for the diagnosis of the pathological side of isolated pure VN. It is a sensitive sign with high negative predictive value.
